# Spliceosomal snRNA Epitranscriptomics

**DOI:** 10.3389/fgene.2021.652129

**Published:** 2021-03-02

**Authors:** Pedro Morais, Hironori Adachi, Yi-Tao Yu

**Affiliations:** ^1^ProQR Therapeutics, Leiden, Netherlands; ^2^Department of Biochemistry and Biophysics, Center for RNA Biology, University of Rochester Medical Center, Rochester, NY, United States

**Keywords:** pre-mRNA splicing, small nuclear RNA, RNA modifications, epitranscriptomics, pseudouridine, 2'-O-methylation, N6-methyladenosine, N2-methylation

## Abstract

Small nuclear RNAs (snRNAs) are critical components of the spliceosome that catalyze the splicing of pre-mRNA. snRNAs are each complexed with many proteins to form RNA-protein complexes, termed as small nuclear ribonucleoproteins (snRNPs), in the cell nucleus. snRNPs participate in pre-mRNA splicing by recognizing the critical sequence elements present in the introns, thereby forming active spliceosomes. The recognition is achieved primarily by base-pairing interactions (or nucleotide-nucleotide contact) between snRNAs and pre-mRNA. Notably, snRNAs are extensively modified with different RNA modifications, which confer unique properties to the RNAs. Here, we review the current knowledge of the mechanisms and functions of snRNA modifications and their biological relevance in the splicing process.

## Introduction

**Figure 1:**
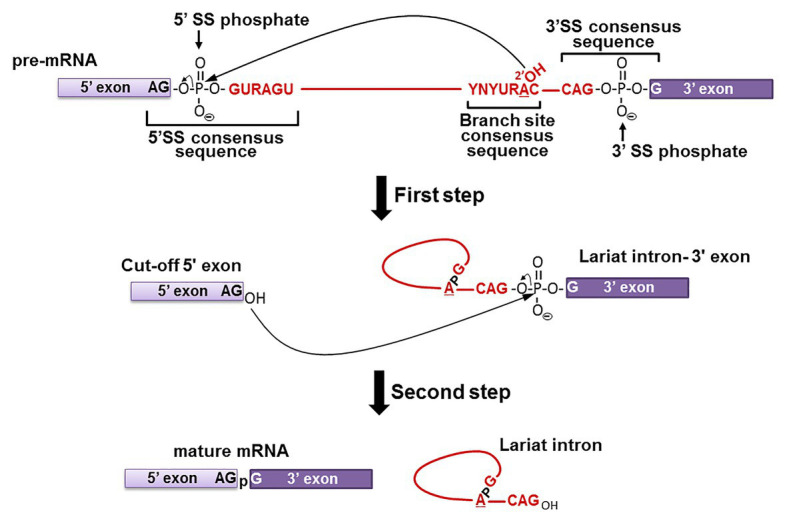
Pre-mRNA splicing pathway. Pre-mRNA splicing takes place *via* a two-step transesterification reaction pathway. In the first step, the 2'-OH group of the branch site adenosine attacks the phosphate at the 5' exon-intron junction (5' SS phosphate), generating the “Cut-off 5' exon” and the “Lariat intron-3' exon” intermediates. In the second step, the 3'-OH group of the “Cut-off 5' exon” attacks the phosphate at the intron-3' exon junction (3' SS phosphate), yielding the “mature mRNA” and “Lariat intron” products. The consensus sequences at the branch site and the 5' and 3' splice sites are shown (red letters, where Y is pyrimidine and R is purine). The 5' exon (light purple box), 3' exon (dark purple box), and the intron (red line) are also shown.

Pre-mRNA splicing is, by definition, a co- or post-transcriptional RNA processing reaction by which introns are removed from mRNA precursors, and exons are precisely joined together to form functional mature mRNAs (Berget et al., 1977; Chow et al., 1977; Shi, 2017). The fidelity of this mechanism is critical for correct gene expression as proven by the fact that 10% of all disease-causing single-point mutations in humans generate splicing defects (Krawczak et al., 2007; Scotti and Swanson, 2016). Pre-mRNA splicing occurs *via* a two-step transesterification reaction pathway (Figure 1; Ruskin et al., 1984). In the first step, the 2'-hydroxyl group (2'-OH) of the branch point nucleotide (adenosine) attacks the phosphate at the 5' exon-intron junction (5' splice site), resulting in the cleavage of the phosphodiester bond between the 5' exon and intron, and the concurrent formation of a new 5'-2' phosphodiester bond between the 5' end of the intron and the branch point adenosine. Thus, a lariat-structured intermediate (lariat intron-3' exon) and a cut-off 5' exon intermediate are produced. In the second step, the 3'-OH group of the cut-off 5' exon attacks the phosphate at the intron-3' exon junction (3' splice site), releasing the lariat intron product and generating the spliced mature mRNA product.

The two chemical reactions of pre-mRNA splicing occur only after the pre-mRNA is assembled into the functional spliceosome, a multi-component complex composed of five small nuclear RNAs (snRNAs U1, U2, U4, U5, and U6), which are present as small nuclear ribonucleoprotein particles (snRNPs, RNA-protein complexes) and a large number of splicing protein factors (Jurica and Moore, 2003).

### Mechanistic Role of snRNA in Pre-mRNA Splicing

**Figure 2:**
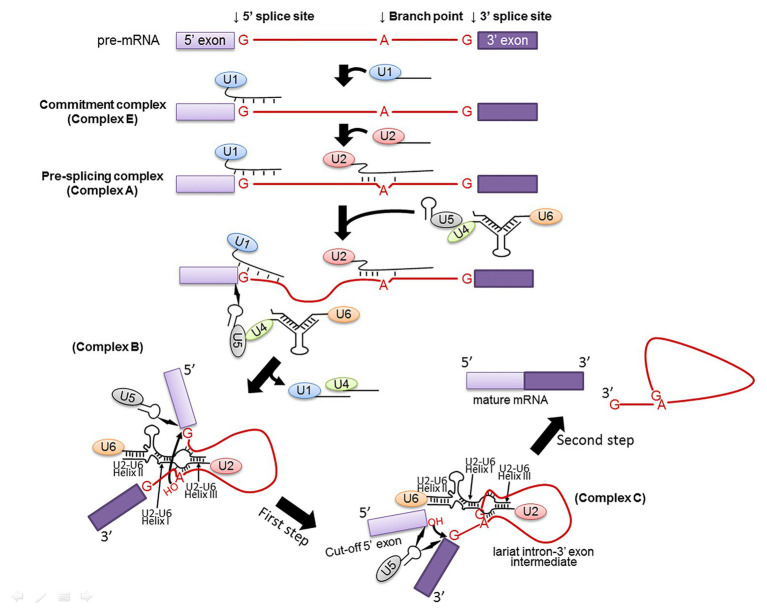
Spliceosome assembly. Spliceosome assembly is a dynamic multi-step process. Shown are several important steps resulting in Complexes E, A, B, and C. The branch site, the 5' and 3' splice sites are also shown. The 5' and 3' exons are in light and dark purple boxes, respectively, and the intron is in red line (and red letters). U1, U2, U4, U5, and U6 snRNPs are also depicted. RNA-RNA interactions, including U1–5' splice site, U2-branch site, U6–5' splice site, U4–U6, and U2–U6 (Helixes I, II, and III) are indicated as well. The two curved arrows indicate nucleophilic attacks (transesterification reactions). The lightning bolts indicate non-Watson-Crick nucleotide-nucleotide contacts.

During spliceosome assembly, spliceosomal snRNPs and splicing factors recognize and interact with the pre-mRNA consensus sequences, facilitating and specifying the transesterification reactions (Figure 2). Specifically, U1 snRNP recognizes the 5' splice site of a pre-mRNA to form a commitment complex (complex E) that commits the pre-mRNA to spliceosome assembly (Kondo et al., 2015). This recognition involves base-pairing interactions between the 10 highly conserved nucleotides at the 5' end of U1 snRNA and the intron sequences of the pre-mRNA at the 5' splice site (G/GUAUGU in yeast or G/GURAGU in vertebrates, where “/” represents the exon-intron junction and R stands for purine; Zhuang and Weiner, 1986). The U2 snRNP then recognizes the pre-mRNA branch site to form a pre-splicing complex called as complex A (Query et al., 1997). This recognition again involves a base-pairing interaction between a highly conserved sequence in U2 snRNA and the pre-mRNA branch site sequence (UACUAAC in yeast or YNYURAC in vertebrates, where Y, R, N, and the underlined adenosine represent pyrimidine, purine, any nucleotide, and the branch point nucleotide, respectively; Parker et al., 1987; Zhuang et al., 1989). While U1 and U2 snRNPs recognize the 5' splice site and the branch site, respectively, the U2 auxiliary factor (U2AF) recognizes the 3' splice site (YAG/G; in vertebrates, the 3' splice site is preceded by a poly-pyrimidine tract; Ruskin et al., 1988; Wu et al., 1999). After the formation of complex A, the U4/U6.U5 tri-snRNP, in which U4 and U6 snRNAs are extensively base-paired, joins this pre-splicing complex, resulting in the formation of a fully assembled spliceosome (complex B; Boehringer et al., 2004; Nguyen et al., 2016; Wan et al., 2016; Plaschka et al., 2017). In the newly formed spliceosome, U5 snRNA associates with the exon sequences at the 5' splice site (*via* non-Watson-Crick nucleotide-nucleotide contact) and possibly interacts with the 3' splice site as well (Newman and Norman, 1992; Wyatt et al., 1992; Sontheimer and Steitz, 1993; Newman et al., 1995; Newman, 1997). Before the first transesterification reaction (first step of splicing) occurs, the spliceosomal RNA-RNA interactions undergo a complex dynamic rearrangement (Wassarman and Steitz, 1992; Nilsen, 1994; Will and Lührmann, 2011). Specifically, U6 snRNA dissociates from U4 snRNA, displaces U1 snRNA in interacting with the 5' splice site (Staley and Guthrie, 1998; Nilsen, 2003), and forms new base-paired duplexes with U2 snRNA (Datta and Weiner, 1991; Wu and Manley, 1991; Madhani and Guthrie, 1992) that are known to be part of the catalytic center (Yean et al., 2000; Wahl et al., 2009; Fica et al., 2013; Zhang et al., 2018). At this point, the first transesterification reaction takes place, leading to the formation of a new complex (complex C), which contains splicing intermediates (Jurica et al., 2004; Will and Lührmann, 2011). After additional conformational changes, the second transesterification reaction (the second step of splicing) occurs, generating matured mRNA and lariat intron products (Will and Lührmann, 2011; Yan et al., 2017). It is important to note that all the interactions occurring in the spliceosome are highly orchestrated, thus allowing for accurate and efficient splicing.

### Epitranscriptomics of snRNAs

The emerging field of epitranscriptomics is continually unveiling additional unknown levels of complexity of the transcriptome (Nachtergaele and He, 2017; Roundtree et al., 2017). RNA modifications can have a major impact in RNA folding and function in all types of RNA, including snRNAs. All snRNAs (except for U6) have a 2,2,7-trimethylated 5' guanosine cap (U6 possesses a γ-monomethyl guanosine cap; Singh and Reddy, 1989). Further, numerous internal nucleotides are modified by pseudouridylation (5-ribosyl isomers of uridine), 2’-O-methylation, and in some cases, base methylation [e.g., *N*^6^-methyladenosine (m^6^A) and *N*^2^-methylguanosine (m^2^G)] (Reddy and Busch, 1988; Massenet et al., 1998; Bohnsack and Sloan, 2018). Notably, the modified nucleotides in spliceosomal snRNAs are remarkably conserved from species to species. For instance, various vertebrate snRNAs contain identical modified nucleotides at identical sites. Although a relatively small number of modified nucleotides have been identified in yeast snRNAs, those that are modified always have counterparts in higher eukaryotic snRNAs (Adachi and Yu, 2014). Furthermore, almost all the modified nucleotides are concentrated in regions that are functionally relevant to the splicing process (Adachi and Yu, 2014). Together, the conservation and the strategic location of these modified nucleotides strongly point to their importance in the process of spliceosome assembly and splicing. It should be pointed out that various post-transcriptional modifications, which are catalyzed by different types of machinery, generate diversity in the snRNAs that likely contribute to pre-mRNA splicing regulation.

In addition to U1, U2, U4, U5, and U6 snRNAs (major), there is also a set of minor spliceosomal snRNA species (U11, U12, U4atac, and U6atac) that participate in the splicing of a minor class of introns (Tarn and Steitz, 1997; Will and Lührmann, 2005; Turunen et al., 2013). Some of these minor snRNAs are also post-transcriptionally modified. This review will describe spliceosomal snRNA modifications (major and minor classes of snRNAs), focusing on the mechanisms and functions of these modifications.

## RNA-Dependent vs. RNA-Independent SnRNA Modification Mechanisms

The most abundant modified nucleotides in snRNAs are pseudouridine (Ψ) and 2'-O-methyl residues, whereas m^6^A and m^2^G are rarely present in only a few snRNA species (Epstein et al., 1980; Reddy et al., 1981b; Massenet et al., 1998; Bohnsack and Sloan, 2018). RNA modification can be catalyzed by either RNA-dependent or RNA-independent mechanism (De Zoysa and Yu, 2017; Meier, 2017; Wiener and Schwartz, 2020). While the RNA-independent mechanism depends on stand-alone protein enzymes capable of recognizing the substrates and catalyzing the chemical reaction, the RNA-dependent mechanism typically relies on RNA-protein enzyme complexes (RNPs), each of which is composed of one small RNA and several proteins. In each RNP, the RNA component functions as a guide recognizing the substrate RNA, and one of the protein components has enzymatic activity catalyzing the chemical reaction (Yu et al., 2005).

### RNA-Dependent Mechanisms

Both snRNA 2’-O-methylation and pseudouridylation are catalyzed by RNA-dependent mechanisms in high eukaryotes. Specifically, a family of box C/D RNPs is responsible for snRNA 2'-O-methylation (Balakin et al., 1996; Cavaillé et al., 1996; Kiss-László et al., 1996), and another family of RNPs, the box H/ACA RNP family, is accountable for snRNA pseudouridylation (Balakin et al., 1996; Ganot et al., 1997; Ni et al., 1997). Each member of the box C/D RNP family is composed of one unique box C/D RNA and four common core proteins (Fibrillarin, also known as Nop1, Nop56, Nop58, and Snu13). Likewise, members of the box H/ACA RNP family each consist of one unique box H/ACA RNA and a set of four common proteins (Dyskerin, also known as Nap57 or Cbf5, Nhp2, Nop10, and Gar1). Both box H/ACA and C/D snoRNAs are usually intron-encoded in mammals and are matured through splicing and processing (Tycowski et al., 1996; Hirose et al., 2003, 2006).

**Figure 3:**
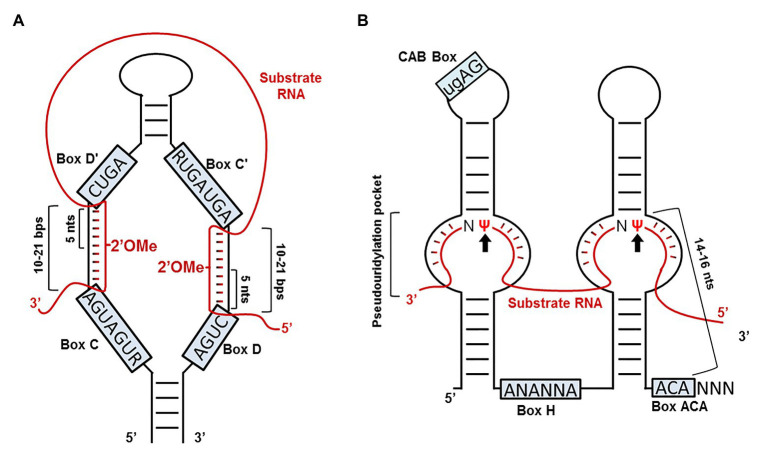
RNA-guided snRNA modifications. **(A)** Box C/D RNA-guided 2'-O-methylation. Box C/D guide RNA (black line) and its substrate RNA (red line) are shown. Boxes C, D', C', and D of the box C/D RNA are indicated. Base-pairing interactions between the two RNAs (usually 10–21 base-pairs) are also depicted. The fifth nucleotide upstream from box D' is 2'-O-methylated (indicated), and so is the fifth nucleotide upstream from box D (also indicated). **(B)** Box H/ACA RNA-guided pseudouridylation. Both box H/ACA RNA (black line) and its substrate RNA (red line) are indicated. Boxes H, ACA, and CAB (a Cajal body localization signal sequence) of the box H/ACA RNA are also indicated. Base-pairing interactions between the guide and substrate RNAs in the pseudouridylation pockets (internal loops) are shown. The modified target uridines (Ψs) are indicated by two arrows. The 14–16 nt distance between the target uridine and box H (or box ACA) is indicated as well.

Mature box C/D RNAs and box H/ACA RNAs both fold into a unique secondary structure (Figure 3). The C/D RNAs, despite their sequence differences, form a signature structure with a terminal stem and two single-stranded sequences sandwiched between box C (RUGAUGA, where R is a purine) and box D' (CUGA) and between box C' (RUGAUGA) and box D (CUGA), respectively (Figure 3). It turns out that the single-stranded sequences serve as guides that base-pair with the substrate RNAs, forming a 10–21-nt duplex and specifying the target nucleotide that is precisely five nucleotides upstream from box D (or box D'). Once the target nucleotide is identified, fibrillarin (Nop1), one of the four box C/D RNP core proteins and a methyltransferase, delivers the methyl group to the target nucleotide at the 2'-O position. The “box D + 5” rule for box C/D RNA-guided snRNA 2'-O-methylation has been verified in various organisms, including *Xenopus*, mouse, and human, indicating that box C/D RNA-guided 2'-O-methylation of snRNA is universal in high eukaryotes (Kiss-László et al., 1996; Karijolich and Yu, 2010). Given that 2'-O-methylation is a sugar-ring modification, it can occur to any nucleotides. Interestingly, to date, no 2'-O-methylated residues have been identified in *Saccharomyces cerevisiae* snRNA.

Similarly, despite their sequence differences, all members of the box H/ACA RNA family fold into a structure known as the “hairpin-hinge (H box, ANANNA, where N represents any nucleotide)-hairpin-tail (ACA box)” structure. In this structure, there are two independent hairpins, in each of which there exists an internal loop (single-stranded) that serves as a guide. In essence, the guide sequences are in two separate segments in the linear RNA that are brought together in internal loops within the hairpins. Base-pairing between the bipartite guide sequence and the snRNA, positions the target uridine at the base of the upper stem of the hairpin, leaving it unpaired within the internal loop (so-called “pseudouridylation pocket”) and located about 14–16 nucleotides upstream of box H or box ACA (Figure 3). When the target uridine is brought to the pocket, Dyskerin (NAP57 or Cbf5), one of the four box H/ACA RNP core proteins and also a pseudouridylase, converts the uridine to pseudouridine. The box H/ACA RNA-guided pseudouridylation mechanism has been tested and verified in various high eukaryotic systems (Terns and Terns, 2006). Recent analyses have further demonstrated that a minimum number of eight base-pairs between the guide and substrate in the pseudouridylation pocket is required for efficient pseudouridylation (Caton et al., 2018; De Zoysa et al., 2018).

In *S. cerevisiae*, spliceosomal snRNA pseudouridylation is more complex. Both RNA-dependent or RNA-independent mechanisms are used (Massenet et al., 1999; Ma et al., 2003, 2005; Yu et al., 2011). The yeast box H/ACA RNAs can be either encoded in introns of protein-coding genes or in independent transcripts of non-protein-coding genes. In *S. cerevisiae*, snRNA pseudouridylation can also be achieved *via* an RNA-independent (protein-only) mechanism.

### RNA-Independent Mechanisms

Stand-alone (protein-only) pseudouridine synthases (Pus) can recognize the substrate and perform the uridine isomerization reaction in a site-specific manner. There are nine Pus enzymes in yeast (Pus1–9) and 11 human homologs (Pus1, Pus3, TruB1, TruB2, RusD1, RusD2, RusD3, RusD4, Pus7, Pus7L, and Pus10; Rintala-Dempsey and Kothe, 2017). In yeast, only Pus1 and Pus7 were identified as capable of RNA-independent pseudouridylation of snRNAs (Massenet et al., 1999; Ma et al., 2003; Basak and Query, 2014; Schwartz et al., 2014). Yeast Pus1 and Pus7 belong to the TruA and TruD families of pseudouridine synthases, respectively. Yeast Pus1 is localized in the nucleus and targets not only snRNAs but also other types of RNA, showing broad substrate specificity (Motorin et al., 1998). In addition to its pseudouridylation activity, Pus1 is also involved in tRNA biogenesis (Simos et al., 1996). Pus7 localizes in the nucleus and cytoplasm of cells and, like Pus1, can also target different RNAs. Pus7 is known for being able to recognize substrates relying on both the sequence (consensus UNUAR motif, where target uridine is underlined) and the secondary structure surrounding the target uridine (Ma et al., 2003; Urban et al., 2009). In yeast, heat shock conditions can further induce the activity of this enzyme (Wu et al., 2011; Schwartz et al., 2014).

Other exotic base methylations, such as m^6^A and m^2^G, have also been identified in snRNAs. These base modifications are catalyzed by RNA-independent enzymatic machineries. Specifically, the m^6^A modification is catalyzed by the m^6^A methyltransferases, protein-only enzymes known as m^6^A writers (Frye et al., 2018). In this reaction, a methyl group is attached to N6 of an adenosine within a specific RNA motif, resulting in the production of m^6^A methylated RNA (RNA containing an N^6^-methyladenosine). m^6^A has been identified in human U2, U4, and U6 snRNAs, as well as in *S. pombe* U2 and U6 snRNAs. While m^6^A writers METTL4 and METTL16 are responsible for the formation of m^6^A in U2 and U6 snRNAs, respectively, the exact enzyme for the formation of m^6^A in human U4 snRNA (Reddy et al., 1981b) remains unknown.

The m^2^G modification results from the methylation of N2 of guanine (the guanosyl amino group at the position C2) and was initially identified in tRNAs (Grosjean et al., 1995). It is catalyzed by a class of enzymes known as guanine-(N2)-methyltransferases, which have been identified in several species (Sindhuphak et al., 1985; Sergiev et al., 2006) and seem to have a substrate consensus sequence (UGGC, the target guanosine is underlined). The m^2^G modification was detected in U6 snRNA decades ago (Epstein et al., 1980), although the nucleotide target sequence in this case (AmGGA, target guanosine is underlined, and the first nucleotide is 2’-O-methylated) deviates from the consensus. In the context of RNA duplexes, this modification is considered as iso-energetic to guanosine (Rife et al., 1998).

## Modified Nucleotides in Spliceosomal SnRNAs

It has long been known that mammalian major spliceosomal snRNAs contain a large number of modified nucleotides (Table 1). Specifically, there are two, fourteen, three, three, and three Ψs in mammalian U1, U2, U4, U5, and U6 snRNAs, respectively. There are also three, ten, four, five, and eight 2’-O-methylated residues in mammalian U1, U2, U4, U5, and U6 snRNAs, respectively. In addition, mammalian U2, U4, and U6 snRNAs each contain an m^6^A. Further, mammalian U6 snRNA contains an m^2^G as well. In contrast, there are a total of only six constitutively formed Ψs in *S. cerevisiae* snRNAs, including two in U1 snRNA, three in U2 snRNA, and one in U5 snRNA. In addition, *S. cerevisiae* U2 snRNA can be pseudouridylated at two novel sites under stress conditions. A set of minor class spliceosomal snRNAs also exists in mammals including U11, U12, U4atac, and U6atac. These snRNAs contain several Ψs and 2'-O-methylated residues as well. Over the years, the mechanisms (what enzymes are involved) and functions of these modifications have been studied, accumulating a wealth of knowledge.

**Table 1:** Yeast and human RNA modifications present in snRNAs with respective guide RNAs (when applicable) and catalyst machinery (Adachi and Yu, 2014).

Species	snRNA	RNA modification	Guide RNA	Catalyst	References
Yeast	U1	Ψ5		Likely non-dependent on box H/ACA mechanism	Massenet et al., 1999
		Ψ6		Likely non-dependent on box H/ACA mechanism	Massenet et al., 1999
	U2	Ψ35	-	Pus7p	Massenet et al., 1999; Ma et al., 2003; Schwartz et al., 2014
		Ψ42	snR81	Cbf5	Massenet et al., 1999; Ma et al., 2005; Schwartz et al., 2014
		Ψ44	-	Pus1p	Massenet et al., 1999; Schwartz et al., 2014
		Ψ56 (stress-induced)	-	Pus7p	Wu et al., 2011
		Ψ93 (stress-induced)	snR81	Cbf5	Wu et al., 2011
	U5	Ψ99		Cbf5p	Massenet et al., 1999; Schwartz et al., 2014
	U6	Ψ28 (filamentous growth induced)		Pus1p	Basak and Query, 2014
Human	U1	Ψ5	ACA47	H/ACA RNP	Branlant et al., 1980; Reddy et al., 1981a; Kiss et al., 2004
		Ψ6	U109	H/ACA RNP	Branlant et al., 1980; Reddy et al., 1981a; Gu et al., 2005
		Am1			Krogh et al., 2017
		Um2			Krogh et al., 2017
		Am70	scaRNA7 (U90)	Fibrillarin	Darzacq et al., 2002; Krogh et al., 2017
	U2	Ψ6		NR	Dönmez et al., 2004; Deryusheva et al., 2012
		Ψ7	U100	H/ACA RNP	Dönmez et al., 2004; Schattner et al., 2006; Deryusheva et al., 2012
		Ψ15		NR	Dönmez et al., 2004; Deryusheva et al., 2012
		Ψ34	scaRNA8 (U92)	H/ACA RNP or Pus7p	Shibata et al., 1975; Darzacq et al., 2002; Kiss et al., 2004; Deryusheva et al., 2012
		Ψ37	ACA45	H/ACA RNP	Shibata et al., 1975; Kiss et al., 2004; Deryusheva et al., 2012
		Ψ39	ACA26	H/ACA RNP	Shibata et al., 1975; Kiss et al., 2004; Deryusheva et al., 2012
		Ψ41	ACA26	H/ACA RNP	Shibata et al., 1975; Kiss et al., 2004; Deryusheva et al., 2012; Deryusheva and Gall, 2018
		Ψ43	scaRNA8 (U92)	H/ACA RNP or Pus1p	Deryusheva et al., 2012; Deryusheva and Gall, 2017
		Ψ44	scaRNA8 (U92)	H/ACA RNP	Shibata et al., 1975; Darzacq et al., 2002; Kiss et al., 2004; Deryusheva et al., 2012
		Ψ54	U93	H/ACA RNP	Shibata et al., 1975; Reddy and Busch, 1988; Kiss et al., 2002, 2004; Schattner et al., 2006; Deryusheva et al., 2012
		Ψ58	SNORA11	H/ACA RNP	Deryusheva et al., 2012, 2020
		Ψ60		NR	Deryusheva et al., 2012
		Ψ89	ACA35	H/ACA RNP	Shibata et al., 1975; Kiss et al., 2004; Deryusheva et al., 2012
		Ψ91		NR	Deryusheva et al., 2012
		Am1			Dönmez et al., 2004; Krogh et al., 2017
		Um2			Krogh et al., 2017
		Gm11	scaRNA2 (HBII-382)	Fibrillarin	Dönmez et al., 2004; Deryusheva et al., 2012; Krogh et al., 2017
		Gm12	SNORD89	Fibrillarin	Dönmez et al., 2004; Deryusheva et al., 2012, 2020; Krogh et al., 2017
		Gm19	scaRNA9 (mgU2–19/30)	Fibrillarin	Dönmez et al., 2004; Deryusheva et al., 2012; Krogh et al., 2017
		Gm25	scaRNA2	Fibrillarin	Deryusheva et al., 2012; Krogh et al., 2017
		Am30	scaRNA9 (mgU2–19/30)	Fibrillarin	Deryusheva et al., 2012; Krogh et al., 2017
		Cm40	MBII-19		Hüttenhofer et al., 2001; Deryusheva et al., 2012; Krogh et al., 2017
		Um47	scaRNA28	Fibrillarin	Deryusheva et al., 2012; Krogh et al., 2017
		Cm61	scaRNA2 (mgU2–25/61)	Fibrillarin	Tycowski et al., 2004; Deryusheva et al., 2012; Krogh et al., 2017
		m^6^Am30		METTL4/Fibrillarin	Mauer et al., 2019; Chen et al., 2020; Goh et al., 2020
	U4	Ψ4		NR	Zerby and Patton, 1997
		Ψ72		NR	Zerby and Patton, 1997
		Ψ79		NR	Zerby and Patton, 1997
		Am1			Krogh et al., 2017
		Gm2			Krogh et al., 2017
		Cm8	scaRNA17 (MBII-119)	Fibrillarin	Krogh et al., 2017
		Am65	scaRNA5 (U87)	Fibrillarin	Darzacq et al., 2002; Krogh et al., 2017
		m^6^A100			Reddy et al., 1981b
	U5	Ψ43	ACA57	H/ACA RNP	Krol et al., 1981; Kiss et al., 2004
		Ψ46	U85	H/ACA RNP	Krol et al., 1981; Jády and Kiss, 2001
		Ψ53	scaRNA13 (U93)	H/ACA RNP	Shibata et al., 1975; Reddy and Busch, 1988; Kiss et al., 2002; Schattner et al., 2006
		Am1			Krogh et al., 2017
		Um2			Krogh et al., 2017
		Gm37			Krol et al., 1981; Krogh et al., 2017
		Um41	scaRNA5/6 (U87)	Fibrillarin	Krol et al., 1981; Darzacq et al., 2002; Krogh et al., 2017
		Cm45	scaRNA10 (U85)	Fibrillarin	Krol et al., 1981; Jády and Kiss, 2001; Darzacq et al., 2002; Krogh et al., 2017
	U6	Ψ31	ACA65	H/ACA RNP	Epstein et al., 1980; Schattner et al., 2006
		Ψ40	ACA12	H/ACA RNP	Epstein et al., 1980; Kiss et al., 2004
		Ψ86	ACA65	H/ACA RNP	Epstein et al., 1980; Schattner et al., 2006; Deryusheva et al., 2020
		Am47	SNORD7 (mgU6–47)	Fibrillarin	Epstein et al., 1980; Tycowski et al., 1998; Krogh et al., 2017
		Am53	SNORD8/9 (mgU6–53)	Fibrillarin	Epstein et al., 1980; Ganot et al., 1999; Krogh et al., 2017
		Gm54			Epstein et al., 1980; Krogh et al., 2017
		Cm60	SNORD67 (HBII-166)	Fibrillarin	Epstein et al., 1980; Hüttenhofer et al., 2001; Lestrade and Weber, 2006; Krogh et al., 2017
		Cm62	SNORD94 (U94)	Fibrillarin	Epstein et al., 1980; Vitali et al., 2003; Krogh et al., 2017
		Cm63			Epstein et al., 1980; Krogh et al., 2017
		Am70			Epstein et al., 1980; Krogh et al., 2017
		Cm77	SNORD10 (mgU6–77)	Fibrillarin	Epstein et al., 1980; Tycowski et al., 1998; Krogh et al., 2017
		m^6^A43		METTL16	Epstein et al., 1980; Shimba et al., 1995; Aoyama et al., 2020
		m^2^G72		Guanine-(N2)-methyltransferases	Epstein et al., 1980
	U12	Ψ19	scaRNA21 (ACA68)	H/ACA RNP	Massenet and Branlant, 1999; Schattner et al., 2006; Deryusheva et al., 2012
		Ψ28	ACA66	H/ACA RNP	Massenet and Branlant, 1999; Schattner et al., 2006; Deryusheva et al., 2012
		Am8			Tycowski et al., 2004; Deryusheva et al., 2012
		Gm18			Tycowski et al., 2004; Deryusheva et al., 2012
		Gm22	scaRNA17 (mgU12–22/U4–8)	Fibrillarin	Darzacq et al., 2002; Tycowski et al., 2004; Deryusheva et al., 2012
	U4atac	Ψ12		NR	Massenet and Branlant, 1999; Deryusheva et al., 2012
		Am1			Deryusheva et al., 2012
		Am2			Deryusheva et al., 2012
		Gm19			Deryusheva et al., 2012
	U6atac	Ψ83	SCARNA21	NR	Massenet and Branlant, 1999; Jorjani et al., 2016

### U1 snRNA

The U1 snRNA is one of the most abundant snRNAs in different species. Only two types of modifications, namely pseudouridylation and 2’-O-methylation, have been detected in mammalian U1 snRNA. Together, there is a total of five modified nucleotides, including Ψ5, Ψ6, Am1, Um2, and Am70 (Reddy et al., 1981a; Reddy and Busch, 1988; Massenet et al., 1999; Kiss et al., 2004; Gu et al., 2005; Krogh et al., 2017; Table 1). In yeast U1 snRNA, only Ψ5 and Ψ6 are identified (Massenet et al., 1999); no 2'-O-methylated residues have been detected. At present, it is still not clear whether the RNA-dependent or RNA-independent mechanism catalyzes pseudouridylation at positions 5 and 6 in yeast U1 snRNA. In human U1 snRNA, pseudouridylation at Ψ5, Ψ6 positions (Branlant et al., 1980) is catalyzed by H/ACA RNP machinery and guided by ACA47 (Kiss et al., 2004) and U109 (Gu et al., 2005), respectively. Mammalian U1 snRNA 2'-O-methylation at position 70 (Krogh et al., 2017) is likely catalyzed by an RNA-dependent mechanism, given that a box C/D RNA (SCARNA7, also known as U90) has been identified to target this site (Darzacq et al., 2002).

#### Functions of Ψs and 2'-O-Methylated Residues Residing in U1 snRNA

Notably, Ψ5 and Ψ6 are within the first 10-nucleotide sequence known to base-pair with the 5' splice site of pre-mRNA during splicing. Given that Ψ can affect local RNA structure and enhance base-pairing and base stacking (Ge and Yu, 2013), Ψ5 and Ψ6 are believed to be important in the recognition process of the 5' splice site. Indeed, an *in vitro* splicing assay performed to test competitive usage of two 5' splice sites suggested that the two Ψs in the U1 snRNA could provide a competitive advantage in the 5' splice site selection (Roca, 2005). In another study, it was shown that Ψ5 or Ψ6 can be bulged out in certain duplexes consisting of 5' splice sites and U1 snRNA (Roca et al., 2012). Thermodynamic analysis of these duplexes confirmed the stabilization properties of Ψs (and 2'-O-methylated residues at the first two positions) in this context, possibly by improving the base stacking of the helix. These results are consistent with the results of a previous work showing that a Ψ in a Ψ-G base pair strengthens the interaction between U1 snRNA and the 5' splice site (Freund, 2003). While these conclusions are exciting and make sense, they seem somewhat contradictory to an earlier work of Will et al. (1996), where the authors showed that U1 snRNA-depleted mammalian cell extracts could still be reconstituted for splicing when adding *in vitro*-transcribed (therefore unmodified) U1 snRNA. However, it could well be that the extracts could modify the *in vitro*-transcribed U1 snRNA upon addition. Alternatively, although the unmodified U1 snRNA could still support splicing, it may not be as active as the modified U1 snRNA. The reconstitution assay using unmodified U1 snRNA probably did not reflect the contributions of Ψ5 and Ψ6 in 5' splice site recognition. As for the function of 2’-O-methylated residue at position 70 (Am70) in mammalian U1 snRNA, not much is known.

### U2 snRNA

U2 snRNA is the most extensively modified among all spliceosomal snRNAs (Shibata et al., 1975; Reddy and Busch, 1988). There are fourteen Ψs, ten 2'-O-methylated residues, and one m^6^Am residue in vertebrate U2 snRNA (Table 1). Given that a near-complete set of box H/ACA RNAs and a complete set of box C/D RNAs are identified and that they can account for almost all known pseudouridylation and 2'-O-methylation sites, it is believed that the RNA-dependent mechanisms are responsible for the formation of virtually all the Ψs and 2'-O-methylated residues (except for the first two 2'-O-methylated residues, Am1 and Um2) in vertebrate U2 snRNA (Hüttenhofer et al., 2001; Tycowski et al., 2004; Schattner et al., 2006; Deryusheva and Gall, 2017, 2018; Bohnsack and Sloan, 2018; Deryusheva et al., 2020). There are three Ψs and no 2'-O-methylated residues identified in *S. cerevisiae* U2 snRNA. The formation of Ψ at different positions within yeast U2 snRNA can be catalyzed by either RNA-dependent or RNA-independent mechanism (Massenet and Branlant, 1999; Ma et al., 2003, 2005; Schwartz et al., 2014).

#### Functions of Ψs and 2'-O-Methylated Residues Residing in Vertebrate U2 snRNA

Many of the U2 snRNA Ψs and 2'-O-methylated residues have been tested for function, and they are virtually all important for splicing. For example, using the *Xenopus* oocyte microinjection system, Yu et al. showed that only modified U2 snRNA (but not *in vitro* transcribed, unmodified U2 snRNA) was able to restore splicing in U2 snRNA-depleted oocytes, indicating that modified nucleotides of U2 snRNA are crucial for pre-mRNA splicing. Subsequently, they mapped the important modified nucleotides to the 5' end region. They further demonstrated that the modified nucleotides within the 5' end region are essential for the formation of functional U2 snRNP and splicing complexes (Yu et al., 1998). In a different study, Dönmez et al. tested these modified nucleotides individually and demonstrated that three Ψs (Ψ6, Ψ7, and Ψ15) and five 2'-O-methylated residues (Am1, Um2, Gm11, Gm12, and Gm19), located in the 5'-end region (first 24 nt) of human U2 snRNA, were required for efficient pre-mRNA splicing. While the Ψs have a cumulative effect in splicing, four of the five 2’-O-methylated residues (Am1, Um2, Gm12, and Gm19) were essential for activity (Dönmez et al., 2004). Soon after, it was shown that the Ψs in the branch site recognition region (BSRR, Ψ34, Ψ37, Ψ39, Ψ41, Ψ43, and Ψ44) are also essential for pre-mRNA splicing (Zhao and Yu, 2004).

#### Functions of Ψs Residing in *Saccharomyces cerevisiae* U2 snRNA

**Figure 4:**
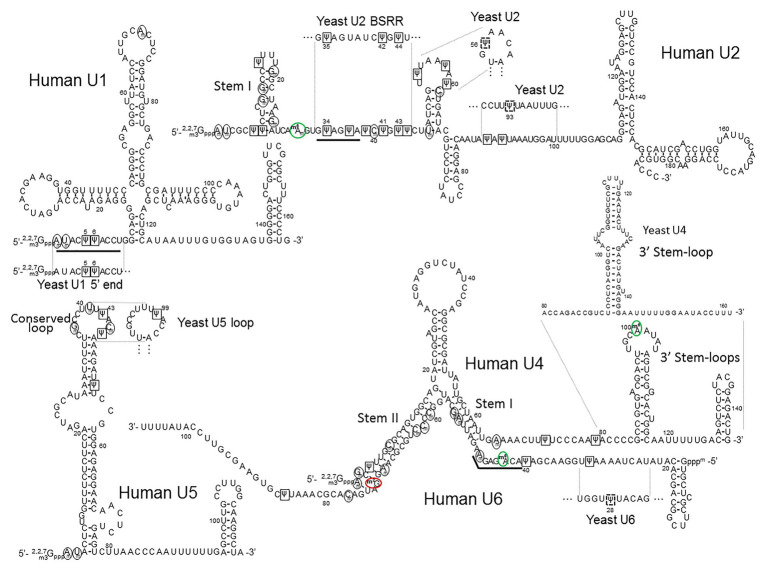
The primary sequences and the secondary structures of major snRNAs. The human U1, U2, U4/U6, and U5 snRNA sequences and structures are shown. The relevant yeast fragments are also depicted. The U2 Stem I, U4 3' Stem-loop, U5 Conserved loop, U4–U6 Stem I and Stem II, are indicated. The sequences that recognize the 5' splice site (the U1 5' end AmUmACΨΨACCU and the U6 ACm^6^AGAGAm sequences) and the branch site (the U2 branch site recognition sequence GΨAGΨA) are underlined. 2'-O-methylated nucleotides are circled (black circles), Ψs are boxed (black squares), inducible Ψs in yeast U2 and yeast U6 are boxed in dashed black squares, m^6^As (including m^6^Am) are circled in green (green circles), and m^2^G is circled in red (red circle).

Unlike vertebrate U2 snRNA, there are only three Ψs that are normally present in *S. cerevisiae* U2 snRNA. They are located at positions 35, 42, and 44 (equivalent to vertebrate U2 snRNA at positions 34, 41, and 43) in the BSRR (Figure 4; De Zoysa and Yu, 2017). Pseudouridylation at these positions, 35, 42, and 44, is catalyzed by Pus7, snR81 RNP, and Pus1, respectively. Among these pseudouridylation enzymes, Pus1 and Pus7 are stand-alone protein pseudouridylases, whereas snR81 RNP is a genuine box H/ACA RNP complex. Several lines of evidence indicate that all these Ψs contribute to branch site recognition during pre-mRNA splicing. For example, Yang et al. showed that a Pus7-deleted strain exhibited reduced levels of splicing and cell growth in certain conditions (Yang et al., 2005). By analyzing splicing in yeast strains deleted of any of the three pseudouridylases (in all combinations), Wu et al. (2016a) found that the three Ψs, in coordination with the ATPase Prp5, play an essential role in recognizing the branch site at an early stage during spliceosome assembly. Furthermore, structural studies of U2 snRNA showed the importance of the Ψ35 in splicing function. Specifically, it was proposed that Ψ35 affected the local RNA structure to expose the branch site adenosine 2'-OH group, making it available for nucleophilic attack on the 5' splice site – the first transesterification reaction or the first step of splicing (Newby and Greenbaum, 2001, 2002). However, recent work from Kielkopf’s lab (Kennedy et al., 2019) suggested that the role of Ψ35 could be indirect, perhaps more reliant on auxiliary factors.

#### Inducible Ψ Formation in *Saccharomyces cerevisiae* U2 snRNA

In yeast U2 snRNA, two non-constitutive modifications (Ψ56 and Ψ93) can also be identified in stress conditions (Wu et al., 2011). Pseudouridylation at positions 56 and 93 is catalyzed, respectively, by Pus7, which normally catalyzes the formation of Ψ35, and snR81 RNP, which is responsible for the constitutive formation of Ψ42. The induction of Ψ formation at these two positions is through the Tor-signaling pathway under nutrient deprivation conditions (Wu et al., 2016b). The formation of Ψ56 can also be induced by heat shock. Although different types of machinery, stand-alone protein Pus7, and box H/ACA RNP snR81, catalyze the formation of Ψ56 and Ψ93, respectively, it appears that the imperfect sequences surrounding the inducible target sites (positions 56 and 93) vs. those flanking the constitutively modified sites (positions 35 and 42) could explain their inducibility (Wu et al., 2011). Induced formation of Ψ56 and Ψ93 plays a role in pre-mRNA splicing, perhaps by helping alter the U2 snRNA structure. Indeed, Ψ56- and Ψ93-mediated U2 snRNA structural change was observed in a structural study (van der Feltz et al., 2018).

#### A Rare Type of Modification (m^6^Am) in Mammalian U2 snRNA

Besides Ψs and 2'-O-methylated residues, one of the 2'-O-methylated adenosines (Am30) in human U2 snRNA is also base methylated in the *N*^6^-position (m^6^Am30; Figure 4). This modification is conserved through evolution, from yeast (*S. pombe*) to humans, in the same nucleotide position (Gu et al., 1996). Since m^6^Am30 is located almost immediately upstream of the branch site recognition sequence, it has recently drawn some attention. Chen et al. (2020) and Goh et al. (2020) have independently identified METTL4 as the methyltransferase responsible for the formation of m^6^Am of mammalian U2 snRNA at position 30. In their study, Chen et al. generated knocked-out METTL4 human cells and observed an effect on splicing in those cells when compared to wild-type cells. However, the direct link between the m^6^Am30 modification in U2 snRNA and splicing was not definitively established. Nonetheless, they demonstrated that METTL4 is the enzyme responsible for the m^6^Am30 modification. Specifically, using recombinant METTL4 and a fragment of U2 snRNA substrate, they carried out an *in vitro* biochemical assay and detected m^6^Am30 formation. However, the 2'-O-methylation of A30 is a prerequisite for the base methylation to occur. Thus, it appears that 2'-O-methylated adenosine (Am30), rather than unmodified adenosine (A30), is the true substrate. Additionally, the level of base methylation could also be severely reduced when changing the 5' and 3' nucleotides, pointing toward sequence recognition by the METTL4. In an independent study, Goh et al. (2020) confirmed METTL4 as the enzyme responsible for the m^6^Am30 modification (with Am being the true substrate). The authors also confirmed the base modification identity with HPLC-MS/MS (Goh et al., 2020). Using transcriptome-wide sequencing, they further showed that this modified nucleotide contributed to splicing regulation. As to the possible mechanism, the authors of this study hypothesized that the modified adenosine (m^6^Am30) could potentially be involved in the recruitment of U2 snRNA to the branch site by U2AF (Zamore et al., 1992; Zhang et al., 1992), a heterodimer that recognizes and binds to the 3' splice site at an early stage of spliceosome assembly (prior to complex A formation), thus affecting the pre-mRNA splicing process (Figure 2).

### U4, U5, and U6 snRNAs

There is also a large number of modified nucleotides in mammalian U4, U5, and U6 snRNAs (Table 1). In total, human U4 snRNA has three Ψs (Ψ4, Ψ72, and Ψ79; Zerby and Patton, 1997), four 2'-O-methylated residues (Am1, Gm2, Cm8, and Am65; Krogh et al., 2017), and one m^6^A (m^6^A100; Reddy et al., 1981b). Human U5 contains several Ψs (Ψ43, Ψ46, and Ψ53; Shibata et al., 1975; Krol et al., 1981) and many 2'-O-methylated residues (Am1, Um2, Gm37, Um41, and Cm45; Krol et al., 1981; Krogh et al., 2017). The human U6 snRNA also has a large number of modified nucleotides, including three Ψs (31, 40, and 86), eight 2'-O-methylated residues (Am47, Am53, Gm54, Cm60, Cm62, Cm63, Am70, and Cm77), one m^6^A (m^6^A43), and one m^2^G (m^2^G72). Most of these modifications were identified decades ago (Epstein et al., 1980; Reddy and Busch, 1988). Similar to mammalian U2 snRNA modifications, pseudouridylation and 2'-O-methylation (except for the first two methylated residues) of human U4, U5, and U6 snRNAs are likely catalyzed by RNA-guided modification mechanisms (Tycowski et al., 1998; Ganot et al., 1999; Hüttenhofer et al., 2001; Jády and Kiss, 2001; Darzacq et al., 2002; Kiss et al., 2002, 2004; Vitali et al., 2003; Lestrade and Weber, 2006; Schattner et al., 2006; Bohnsack and Sloan, 2018). Interestingly, there is only one Ψ (Ψ99) and no 2'-O-methylated nucleotide in *S. cerevisiae* U5 snRNA (Massenet et al., 1999). No Ψ nor 2'-O-methylated residues were identified in yeast U4 and U6 snRNAs under normal growth conditions.

#### Functions of Ψs and 2'-O-Methylated Residues Residing in U4 and U6 snRNAs

While the function of Ψs and 2'-O-methylated residues in yeast and mammalian U4, U5, and U6 snRNAs remains largely unclear, it is speculated that these modifications play a crucial role in splicing. Before participating in spliceosome assembly, U4, U5, and U6 snRNAs assemble into the U4/U6.U5 tri-snRNP particle, in which U4 and U6 snRNAs form an extensive base-pair interaction. The strength of this interaction was empirically determined as a stable one (Brow and Guthrie, 1988). Because of their presence in the base-paired region, Ψs and 2'-O-methylated residues seem to be particularly relevant (Figure 4). Given that Ψs and 2'-O-methylated residues are known to increase base-stacking and enhance base-pairing, it is possible that these modified nucleotides in the U4–U6 helixes contribute to stabilizing the interaction. However, the base-pairing between U4 and U6 snRNAs must eventually unwind for the catalytically active spliceosome to form after the U4/U6.U5 tri-snRNP particle enters complex A (pre-mRNA complexed with U1 and U2 snRNPs; see Figure 2). This unwinding event is performed by Brr2, an ATP-dependent helicase with two helicase cassettes in tandem, although only the N-terminal one has unwinding activity (Raghunathan and Guthrie, 1998). Nguyen et al. (2015) obtained the cryo-EM structure of U4/U6.U5 tri-snRNP in yeast and observed that the Brr2 active site is preloaded in the single-stranded region between the stem I of the U4-U6 duplex region and the 3' stem-loop of the U4 snRNA (Figure 4). It is speculated that, since the human U4 snRNA has two Ψs in this single-stranded region (Ψ72 and Ψ79), they could be involved in the recruitment of (or recognition by) the helicase. However, upon deciphering the structure of human U4/U6.U5 tri-snRNP (also by cryo-EM), Agafonov et al. were able to find that Brr2 is located in a different position within the human U4/U6.U5 tri-snRNP complex, approximately 8–10 nm away from the U4/U6 snRNA duplex (Agafonov et al., 2016). To understand the function of Ψ72 and Ψ79 of human U4 snRNA, further research is necessary.

In the course of activation of the spliceosome, or after the unwinding of the U4/U6 snRNA duplex, U1 and U4 snRNAs leave the spliceosome, and the U2, U5, and U6 snRNAs interact with pre-mRNA and with each other (see Figure 2). In particular, U2 and U6 snRNAs form three short base-paired duplexes (helixes I, II, and III; Datta and Weiner, 1991; Madhani and Guthrie, 1992; Sun and Manley, 1995; Burke et al., 2012), which are believed to be the catalytic center for splicing (transesterification) reactions. The dynamic formation of U2–U6 snRNA duplexes was also studied in a protein-free system, where U2–U6 snRNA interactions (likely related to the spliceosomal U2–U6 snRNA helixes) were detected (Burke et al., 2012; Chu et al., 2020). Notably, there are multiple modified nucleotides (Ψ and 2’-O-methylated residues) in the U2-U6 snRNA duplexes. Using single-molecule fluorescence, Karunatilaka and Rueda further investigated the role of these modified nucleotides in the dynamics of U2-U6 snRNA interactions. They concluded that the modifications present in the U2 snRNA stem I (Figure 4) contribute to the dynamics and conformation of the U2-U6 snRNA complex (Karunatilaka and Rueda, 2014). They also suggested that the modified nucleotides in the U2–U6 snRNA complex might also contribute to protein binding in addition to direct RNA structure stabilization.

#### Functions of Ψs and 2'-O-Methylated Residues Residing in U5 snRNA

In the spliceosome, U5 snRNA interacts, through its conserved loop, with the pre-mRNA by directly contacting (non-Watson-Crick pairing) the exon nucleotides at both the 5' and 3' splice sites. Notably, there are two Ψs and three 2'-O-methylated residues in this conserved loop sequence (GmCCUUmU*Ψ*ACm*Ψ*) of human U5 snRNA (Frank et al., 1994; Szkukalek et al., 1995). In *S. cerevisiae* U5, however, there is only one modified nucleotide (Ψ99), but it is located in the same conserved loop sequence (GCCUUUΨAC; Figure 4). However, it should be noted that despite the conservation, there is not yet direct evidence indicating that these modified nucleotides contribute to the U5-pre-mRNA interactions.

#### Other Types of Modifications in U6 snRNA

It was recently reported that METTL16 functions as the methyltransferase responsible for the formation of m^6^A at position 43 within mammalian U6 snRNA (Pendleton et al., 2017; Warda et al., 2017; Aoyama et al., 2020). This modification (m^6^A43; Shimba et al., 1995) could have a direct role in splicing regulation given that it is conserved from *S. pombe* (at position 37; Gu et al., 1996) to human and that it is located in the region which forms base-pairing interactions with the 5' splice site of pre-mRNA before the first step of splicing reaction (transesterification reaction) occurs (Figures 2, 4). In this regard, it has been shown that mutations in this region (ACAGAGA), where m^6^A43 (underlined) is located, can be lethal in the yeast organism (Madhani et al., 1990). While these mutations might have directly disrupted the interaction between U6 snRNA and the 5' splice site of pre-mRNA leading to lethality, it is also possible that the mutations prevented the formation of m^6^A43, which is potentially important for 5' splice site recognition in *S. pombe* and higher eukaryotes. Further work is necessary to elucidate the role of m^6^A43. Mammalian U6 snRNA also contains the m^2^G at position 72 (Epstein et al., 1980). While the exact function of m^2^G72 in U6 snRNA is still largely unclear, this modified nucleotide is known to base-pair with C3 of U4 snRNA in the U4–U6 duplex structure (see Figure 4). Since this modification was determined empirically to have the same thermodynamic stability as unmodified guanosines in the context of a G:C base pair (Rife et al., 1998), one could speculate that its role in splicing could potentially be related to recognition by a splicing factor.

#### Inducible Ψ Formation in *Saccharomyces cerevisiae* U6 snRNA

Another study was carried out in the Query lab, focusing on the inducible pseudouridylation of *S. cerevisiae* U6 snRNA at position 28 (Ψ28; Figure 4). They showed that the formation of Ψ28 occurred under certain filamentous growth conditions. This growth condition-induced pseudouridylation is catalyzed by Pus1. Subsequent analyses allowed the authors to conclude that Ψ28 in U6 snRNA directly contributes to filamentous formation (Basak and Query, 2014).

### Minor Spliceosomal snRNAs

**Figure 5:**
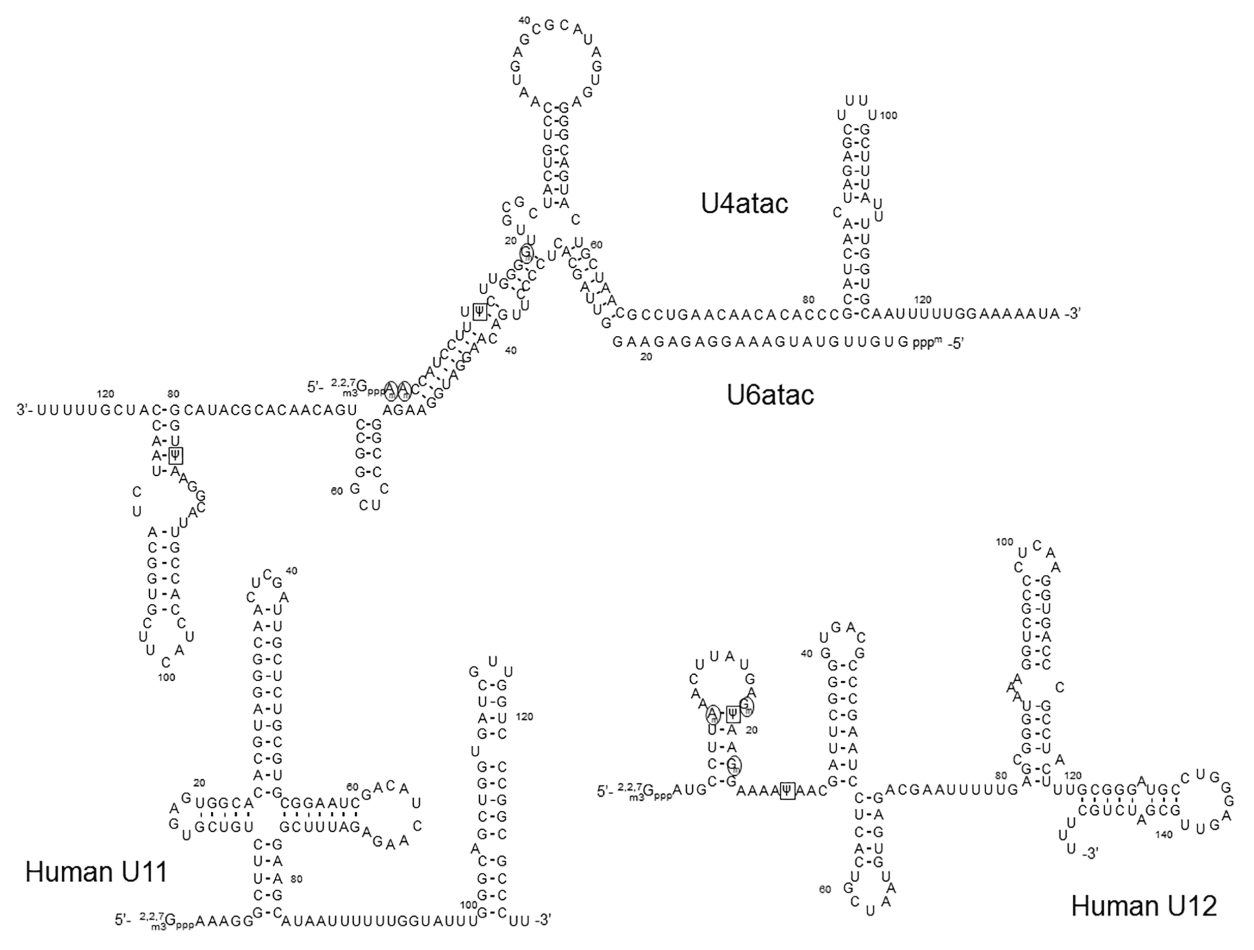
The primary sequences and the secondary structures of minor class snRNAs. The sequences and secondary structures of human U11, U12, U4atac, and U6atac snRNAs are shown. The sequences that recognize the 5' splice site of minor class introns (the U11 5' end and the U6atac sequences) and the branch site (the U12 branch site recognition sequence) are underlined. 2'-O-methylated nucleotides are circled (black circles), Ψs are boxed (black squares),

In addition to the major spliceosomal pathway (U2-dependent, described above), there is a less common (or minor) pathway required for splicing of rare class introns that contain different consensus sequences at the 5' and 3' splice sites and the branch site. Except for U5, which is common for both major and minor splicing pathways, a different set of spliceosomal snRNAs (U11, U12, U4atac, U6atac, and U5) is required for the minor splicing pathway (Figure 5). Because it depends on U12 (rather than U2), the minor splicing pathway is also known as the U12-dependent splicing pathway (Montzka and Steitz, 1988; Tarn and Steitz, 1996; Turunen et al., 2013). In the U12-dependent splicing pathway, U11, U12, U4atac, and U6atc snRNAs each play a role that is equivalent to the role of U1, U2, U4, and U6 snRNAs in the major spliceosome, respectively. Expectedly, the secondary structures of the minor class snRNAs are very similar to those of their major class snRNA counterparts (Figure 5). RNA-guided nucleotide modifications (pseudouridylation and 2'-O-methylation) in the minor-class snRNAs have also been studied (Jorjani et al., 2016).

In an attempt to map Ψs in the minor spliceosome snRNAs in HeLa cells, Massenet and Branlant performed the pseudouridylation assay (CMC-modification followed by primer-extension) and identified Ψs in U12, U4atac, and U6atac snRNAs (Massenet and Branlant, 1999). Surprisingly, no Ψ was detected in U11 snRNA, although its major spliceosome counterpart U1 snRNA has two Ψs located in the 5'-end region that base-pairs with the 5'-splice site. It should be noted, however, that the 5' 10-nucleotide sequence (AAAAAGGGCU) of U11 snRNA (equivalent to the 5' 10-nucleotide sequence of U1 snRNA, AUACΨΨACCU) lacks the two U-residues (to be pseudouridylated) at the equivalent positions of U1 snRNA (Yu and Steitz, 1997). In the U12 snRNA, the authors detected only two Ψs at positions 19 and 28. Ψ19 is equivalent to Ψ34 of mammalian U2 snRNA that base-pairs with the nucleotide (A or G) within the branch site sequence that is immediately next to the bulged-out nucleotide (branch point adenosine), pointing toward a functional role of this Ψ. Ψ28 is located in a region that forms a base-pairing helix III with U6atac (equivalent to U2-U6 helix III), which has been shown to have a functional role in the splicing of U2-dependent introns (Sun and Manley, 1995). In the U4atac snRNA, a single Ψ was detected at position 12, located in the region that base-pairs with U6atac, equivalent to U4-U6 stem II in which there are several modified nucleotides in the U4 strand. Finally, Ψ83 was also identified in the U6atac snRNA 3'-end region. This Ψ could be functionally similar to Ψ86 in the U6 snRNA 3' terminal region. In a later study, these Ψs were all confirmed (Deryusheva et al., 2012). In addition, several 2'-O-methylations were also identified in U12 (at positions 8, 18, and 22) and U4atac snRNAs (at positions 1, 2, and 19, and potentially also position 8, although yet to be confirmed; Deryusheva et al., 2012). However, the exact function of these modified nucleotides remains unknown.

## Concluding Remarks

Understanding the splicing mechanisms at the molecular level is of critical importance not only to fully comprehend gene expression but also to develop new nucleic acid-based therapeutics, such as splice-switching oligonucleotides (Lim and Yokota, 2018), aimed at correcting splicing-associated mutations that lead to aberrant proteins and diseases. pre-mRNA splicing occurs in the spliceosome, an extremely large complex consisting of five snRNAs and a large number of proteins that interact with substrate pre-mRNA in a highly orchestrated manner. These snRNAs have a critical role in guiding the overall process *via* base-pairing interactions (and nucleotide-nucleotide contact) with the substrate pre-mRNA. Additionally, the snRNAs form dynamic structures that might be crucial for protein recruitment and catalysis. Post-transcriptionally modified nucleotides might contribute significantly in each of these steps during spliceosome assembly and splicing.

snRNA modifications, such as pseudouridine and 2'-O-methylation, have attracted a great deal of attention over the years, and extensive studies of these modifications have provided valuable insights into the mechanism of pre-mRNA splicing regulation. The continuous increase of knowledge of the fine-tuning and subtleties provided by RNA modifications in the spliceosome assembly and splicing processes are benefiting the development of better splicing modulation technologies. With the growing number of clinical trials based on splicing modulation therapies (exon-skipping or exon-inclusion) and the FDA-approved drugs based on this mechanism of action (Stein and Castanotto, 2017; Rüger et al., 2020), the interest in this field will certainly continue to grow. The novel deep sequencing chemical probing technologies and epitranscriptomics analytical techniques will help us to decipher the yet-to-be discovered code of spliceosomal RNA modifications.

## Author Contributions

PM, HA, and Y-TY wrote the manuscript and generated the figures. All authors read and approved the final manuscript.

### Conflict of Interest

PM is a scientific director at ProQR Therapeutics.

The remaining authors declare that the research was conducted in the absence of any commercial or financial relationships that could be construed as a potential conflict of interest.
